# 高效液相色谱联合2,2'-二硫二吡啶衍生反应测定人血清不同类型游离巯基及其与冠心病的关系分析

**DOI:** 10.3724/SP.J.1123.2024.02001

**Published:** 2024-04-08

**Authors:** Zhiyu SHAO, Jie ZENG, Jun DONG, Hongxia LI, Ruiyue YANG, Wenxiang CHEN, Siming WANG

**Affiliations:** 北京医院 国家老年医学中心, 国家卫生健康委北京老年医学研究所, 国家卫生健康委老年医学重点实验室, 中国医学科学院老年医学研究院, 北京 100730; The Key Laboratory of Geriatrics, Beijing Institute of Geriatrics, Institute of Geriatric Medicine, Chinese Academy of Medical Sciences, Beijing Hospital/National Center of Gerontology of National Health Commission, Beijing 100730, China

**Keywords:** 高效液相色谱, 衍生反应, 2,2'-二硫二吡啶, 游离巯基, 血清, 冠心病, high performance liquid chromatography (HPLC), derivatization reaction, 2,2'-dithiodipyridine, free thiols, serum, coronary heart disease

## Abstract

游离巯基对于心血管疾病的诊断和治疗具有潜在的临床预示价值,本研究建立了一种基于2,2'-二硫二吡啶衍生反应的高效液相色谱测定人血清游离巯基的分析方法,能够同时获得人血清中总游离巯基(Total-SH)、小分子化合物形态游离巯基(LMM-SH)、蛋白质形态游离巯基 (P-SH)三者的含量。选用Agilent Eclipse XDB-C18色谱柱(150 mm×4.6 mm, 5 μm),以0.1%甲酸水溶液-0.1%甲酸乙腈溶液为流动相,在1 mL/min的流速下进行梯度洗脱,5 min内可实现良好的色谱峰基线分离,其中2-硫代吡啶酮色谱峰代表血清样本中总游离巯基含量,吡啶二硫衍生物色谱峰代表小分子化合物形态游离巯基含量,二者差值为蛋白质形态游离巯基含量。本研究对衍生反应条件进行了优化,并进行了方法学验证,结果表明,该方法线性关系良好,相关系数≥0.9994,线性范围为31.25~1000 μmol/L,Total-SH和LMM-SH的检出限分别为2.61 μmol/L和0.50 μmol/L,定量限分别为8.71 μmol/L和1.67 μmol/L;加标回收率为91.1%~106.0%,日内精密度和日间精密度为0.4%~9.1%。使用本方法测定了714名志愿者的血清样本,总游离巯基浓度为376.60~781.12 μmol/L,平均浓度为555.62 μmol/L;小分子化合物形态游离巯基的浓度为36.37~231.65 μmol/L,平均浓度为82.34 μmol/L;蛋白质游离巯基浓度为288.36~687.74 μmol/L,平均浓度为473.27 μmol/L。Spearman相关性检验分析发现,血清游离巯基浓度与冠心病严重程度及常见临床生化指标存在密切关联。本研究提供了一种简便可靠的血清游离巯基分析方法,探索了其与冠心病的关系,为冠心病风险相关标志物的研究提供了新的参考指标。

## Abstract (en)

Oxidative stress, which is characterized by an imbalance between antioxidants and free radicals, plays a pivotal role in the pathogenesis of coronary heart disease, a common and serious cardiovascular condition, and contributes significantly to its development and progression. Serum free thiols are crucial components of the body’s antioxidant defense system. The accurate determination of serum free thiol levels provides a reference basis for understanding the body’s status and monitoring the risk factors associated with the occurrence and progression of coronary heart disease. In this study, a high performance liquid chromatographic (HPLC) method based on the derivatization reaction of 2,2'-dithiodipyridine was developed to simultaneously obtain the concentrations of total free thiols (Total-SH), low-molecular-mass free thiols (LMM-SH), and protein-free thiols (P-SH) in human serum. An Agilent Eclipse XDB-C18 column (150 mm×4.6 mm, 5 μm) was used for the analysis, and gradient elution was performed at a flow rate of 1 mL/min. A 0.1% formic acid aqueous solution was used as mobile phase A, and a 0.1% formic acid acetonitrile solution was used as mobile phase B. The gradient elution program was as follows: 0-0.1 min, 12%B-30%B; 0.1-2 min, 30%B; 2-2.1 min, 30%B-100%B; 2.1-6 min, 100%B; 6-6.1 min, 100%B-12%B; 6.1-7 min, 12%B. Well-separated peaks appeared after a run time of 5 min. The peak of 2-thiopyridone represented the Total-SH content of the samples, and the peak of the pyridyldithio derivative represented the LMM-SH content. The difference between these two peaks indicated the P-SH content. The derivatization reaction conditions were optimized, and the method was validated. The method demonstrated good linearity, with a correlation coefficient ≥0.9994, over the concentration range of 31.25-1000 μmol/L. The limits of detection for Total-SH and LMM-SH were 2.61 and 0.50 μmol/L, and the limits of quantification for Total-SH and LMM-SH were 8.71 and 1.67 μmol/L, respectively. The recoveries of Total-SH and LMM-SH were in the range of 91.1%-106.0%. The intra- and inter-day precisions ranged from 0.4% to 9.1%. The developed method was used to analyze serum samples from 714 volunteers. The Total-SH concentrations ranged from 376.60 to 781.12 μmol/L, with an average concentration of 555.62 μmol/L. The LMM-SH concentrations varied from 36.37 to 231.65 μmol/L,with an average of 82.34 μmol/L. The P-SH concentrations ranged from 288.36 to 687.74 μmol/L, with an average of 473.27 μmol/L. Spearman’s correlation test showed that serum thiol levels were correlated with the severity of coronary artery disease and common clinical biochemical indicators. The proposed study provides a simple and reliable HPLC method for detecting serum free thiols and exploring their relationship with coronary heart disease, offering a new reference for the study of markers related to the risk of coronary heart disease.

冠状动脉粥样硬化性心脏病(冠心病)是全球范围内危害人类健康的严重疾病之一,同时也是我国城乡居民的第一死因,且发病年轻化趋势日益明显^[1]^。冠心病病理过程复杂,其中氧化应激被认为是促进动脉粥样硬化发生发展的重要因素^[2]^。它能导致活性氧(ROS)的过度产生和积累,从而直接损伤血管内皮细胞,造成动脉硬化;另外氧化应激还会促进炎症细胞浸润和血小板聚集,导致血管斑块形成,引发心血管事件发生^[3]^。因此,尽可能消除氧化应激是预防冠心病、降低疾病危害的重要途径之一^[4]^。

游离巯基具有抗氧化作用,是人体对抗氧化应激的重要生物活性分子^[5]^。总游离巯基(Total-SH)主要由蛋白质游离巯基(P-SH)和小分子化合物形态游离巯基(LMM-SH)两部分组成。其中蛋白质表面的游离巯基一般占血清Total-SH的80%,而血清中的LMM-SH主要包括半胱氨酸、半胱氨酰甘氨酸、谷胱甘肽、同型半胱氨酸和*γ*-谷氨酰半胱氨酸等多种化合物^[6]^。已有临床研究表明血清游离巯基水平与冠心病的严重程度密切相关,其可作为早期独立预测冠心病的指标^[7]^。Abdulle等^[8]^证明血清游离巯基水平与人群心血管事件和全因死亡率两者呈明显的负相关趋势。Cheraghi等^[9]^研究发现冠心病患者的血清谷胱甘肽水平显著低于健康人群。这些结果说明游离巯基对于心血管疾病的诊断和治疗具有潜在的临床预示价值。建立人血清游离巯基准确测定方法并用于人群样本游离巯基水平监测,有利于辅助冠心病早期诊断和预后评估,具有重要的社会和科学意义。

目前针对游离巯基的检测方法包括酶联免疫吸附法^[10]^、光度法^[11]^、电化学方法^[12]^等。虽然这些方法具有简单、低成本等优点,但是它们对复杂生物样本的适用性相对较低,特别是仅能测得游离巯基总量,无法对不同来源的游离巯基进行区分。与这些方法相比,高效液相色谱法(HPLC)作为一种能够分离复杂体系、可同时对多组分进行定量、易与各种检测器联用的分析方法,在生物样本游离巯基测定分析中具有潜在优势^[13,14]^。但需要指出的是由于一般巯基化合物不发光且不具备生色团,因此需要对游离巯基进行衍生化处理,利用衍生增色后的产物光学信号对游离巯基进行检测。同时,游离巯基本身不稳定,也需要先通过衍生化反应将其转化为稳定化合物后才能进行准确定量。

针对上述情况,本研究建立了一种基于2,2'-二硫二吡啶(2,2'-DTDP)衍生反应的HPLC测定人血清游离巯基的分析方法。本方法简便可靠,可以在一个分析周期内同时获得样本中Total-SH、LMM-SH、P-SH三者的含量。在此基础上,我们应用所建方法研究了临床志愿者血清中游离巯基的含量及分布情况,探索了游离巯基与冠心病及心血管事件之间的关联,为临床研究游离巯基与冠心病的关系提供了更加丰富、客观的参考依据。

## 1 实验部分

### 1.1 仪器、试剂与材料

#### 1.1.1 仪器与试剂

Agilent 1200高效液相色谱仪(美国Agilent公司),配备Agilent Chemstation软件进行仪器控制和数据采集。半胱氨酸、半胱氨酰甘氨酸、谷胱甘肽、同型半胱氨酸、*γ*-谷氨酰半胱氨酸、2,2'-DTDP均购自美国Sigma-Aldrich公司。色谱纯级别的甲酸、乙腈、甲醇购自美国Fisher Scientific公司。十二水合磷酸氢二钠和二水合磷酸二氢钠购自阿拉丁公司。乙二胺四乙酸二钠购自福晨化学公司。七水合硫酸锌购自比利时ACROS公司。实验中使用的其他化学试剂均为分析纯。去离子水由Milli-Q超纯水仪(美国Millipore公司)制备。

#### 1.1.2 血清样本

征集714名北京医院心内科行冠状动脉造影的志愿者(年龄19~91岁,男性402名,女性312名),采集静脉血于采血管中,在4 ℃下以3500 r/min离心10 min,取出上层的血清分装于冻存管中,置于-80 ℃保存。临床常规生化检验指标部分由临床检验数据系统直接调取,部分由本实验室既往研究测得^[15,16]^。本研究获得北京医院医学伦理委员会的批准(2016BJYYEC-121-05),志愿者均签署知情同意书。

### 1.2 实验方法

#### 1.2.1 标准溶液、衍生试剂溶液配制

以超纯水为溶剂,配制浓度为25 mmol/L的半胱氨酸溶液作为储备液,将储备液依次稀释,配制成浓度为31.25、62.5、125、250、500、1000 μmol/L的游离巯基标准溶液,分装于冻存管中,于-80 ℃避光储存。以乙腈为溶剂,配制浓度为20 mmol/L的2,2'-DTDP。

#### 1.2.2 样本制备

将血清样本、标准溶液取出解冻、混匀并恢复至室温。吸取200 μL标准溶液或血清样品于安瓿瓶中,加入200 μL磷酸盐缓冲溶液(0.1 mol/L, pH 7)和100 μL 20 mmol/L 2,2'-DTDP溶液,涡旋混匀后于室温(25 ℃)下避光孵育5 min,加入400 μL硫酸锌水溶液(1 mol/L)与甲醇的混合溶液(1∶1, v/v)沉淀样本中的蛋白质,混匀后于4 ℃下以3500 r/min离心10 min,吸取200 μL澄清上清液,转移至自动进样小瓶中,随后进行HPLC分析。

#### 1.2.3 色谱条件

采用Agilent Eclipse XDB-C18色谱柱(150 mm×4.6 mm, 5 μm)。流动相A相为0.1%甲酸水溶液,B相为0.1%甲酸乙腈溶液。以1 mL/min的流速进行梯度分离,梯度如下:0~0.1 min, 12%B~30%B; 0.1~2 min, 30%B; 2~2.1 min, 30%B~100%B; 2.1~6 min, 100%B; 6~6.1 min, 100%B~12%B; 6.1~7 min, 12%B。柱温25 ℃,进样体积50 μL,检测波长280 nm。

### 1.3 血清样本测定

用本研究建立的方法测定714位志愿者的血清巯基含量,使用SPSS 25统计软件进行数据统计分析。采用Spearman相关性检验分析血清巯基含量与冠心病及常见临床生化指标的关系。定义*P*<0.05为具有统计学显著性意义。

## 2 结果与讨论

### 2.1 衍生反应体系的选择

目前常用的巯基衍生剂5,5'-二硫代双(2-硝基苯甲酸)对pH值较敏感,过酸或过碱都会导致衍生反应进行不完全或衍生剂自身分解^[17]^。2,2'-DTDP可与游离巯基在酸性或中性条件下发生巯基-二硫键交换反应生成产物硫代吡啶酮(2-TP)和吡啶二硫衍生物(图1)^[18]^,衍生剂和衍生产物均在280 nm波长下具有良好的紫外吸收,可以实现衍生底物和产物的同时监测^[19]^,因此本研究选用2,2'-DTDP衍生体系。样本沉淀蛋白后进行色谱分析,上清液中的2-TP浓度代表了血清样品中总游离巯基含量,吡啶二硫衍生物浓度则反映样本中小分子化合物形态游离巯基总量。以Total-SH浓度减去LMM-SH浓度,即可得到血清中P-SH含量。

**图 1:**
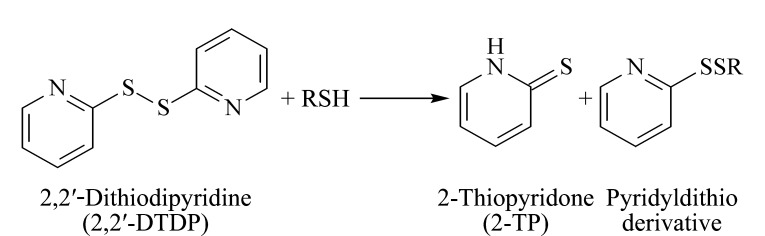
游离巯基与2,2'-DTDP的衍生反应方程式

### 2.2 色谱条件优化

#### 2.2.1 色谱柱

本研究考察了两根色谱柱,分别为Waters Symmetry Shield RP18色谱柱(250 mm×4.6 mm, 5 μm)和Agilent Eclipse XDB-C18色谱柱(150 mm×4.6 mm, 5 μm)。结果发现,相对于前者,后者能获得更好的分离度,同时峰形更尖锐,响应信号更好,保留时间更合适。故选用Agilent Eclipse XDB-C18色谱柱(150 mm×4.6 mm, 5 μm)。

#### 2.2.2 流动相

流动相A使用纯水,流动相B使用乙腈。在流动相中添加0.1%甲酸以抑制溶质离子化、减少色谱峰拖尾。改变流动相的比例和洗脱程序使色谱峰能够在适宜的保留时间内得到有效分离。鉴于单种LMM-SH在人血清中含量均较低、不易检测,同时LMM-SH的总体水平相较于单种更能有效地反映机体对抗氧化应激的整体能力,故本研究对流动相梯度进行优化,使多种LMM-SH色谱峰的保留时间一致,形成一个能够反映LMM-SH含量的色谱峰,即小分子巯基吡啶衍生物(LMM-S-S-Pyr)色谱峰(见图2)。此外2,2'-DTDP与色谱柱相互作用较强,在保证各色谱峰良好分离基础上需使2,2'-DTDP尽快洗脱出峰以缩短方法运行时间,提高分析效率。综合考虑,最终的流动相条件确定为流动相A相为0.1%甲酸水溶液,B相为0.1%甲酸乙腈溶液,以1 mL/min的流速进行梯度洗脱色谱分离,梯度条件见1.2节。

**图 2:**
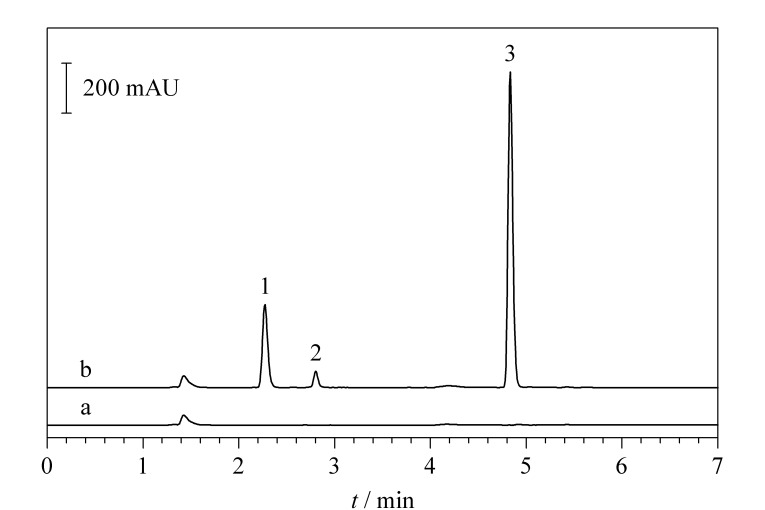
血清样本使用2,2'-DTDP衍生前后的色谱图

在优化的色谱条件下,血清样本的色谱图如图2b所示,5 min内全部待测物均洗脱出峰,依次为2-TP、LMM-S-S-Pyr、2,2'-DTDP,各色谱峰峰形良好,能够实现基线分离。本研究所建方法一次进样即可同时获得样本中Total-SH、LMM-SH、P-SH三者的含量,提高了分析效率。

### 2.3 样品前处理条件的优化

#### 2.3.1 衍生试剂浓度

本研究分别考察了5、10、20、40、60 mmol/L 2,2'-DTDP溶液对血清样本游离巯基衍生效率的影响。结果如图3a所示,2,2'-DTDP溶液浓度为5~20 mmol/L时,2-TP峰面积随2,2'-DTDP浓度的升高而增大,继续增加2,2'-DTDP浓度时待测物峰面积基本不变。表明衍生试剂浓度选择20 mmol/L最为合适,既可确保充分反应又避免过量加入2,2'-DTDP衍生剂引起未知干扰。

**图 3:**
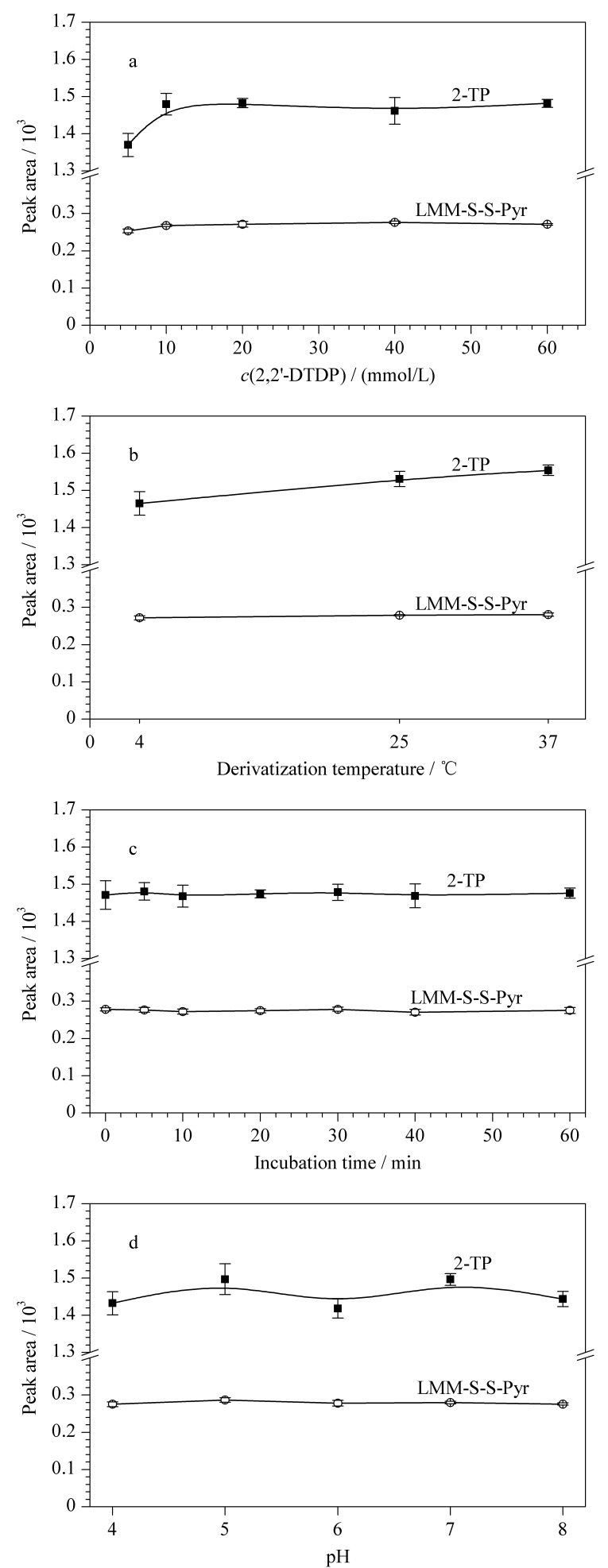
(a)衍生试剂浓度、(b)反应温度、(c)反应时间、 (d)缓冲液pH对待测物峰面积的影响(*n*=3)

#### 2.3.2 反应温度

分别考察在4 ℃、25 ℃以及37 ℃条件下进行衍生反应对血清样本游离巯基衍生效率的影响。结果如图3b所示,25 ℃以及37 ℃条件下的衍生效率均较4 ℃略有升高,25 ℃与37 ℃条件相比两者未见显著变化。反应温度最终选择25 ℃。

#### 2.3.3 反应时间

考察衍生反应时间对游离巯基衍生反应的影响。结果发现衍生反应时间的长短对衍生产物浓度无明显影响(图3c)。综合考虑,选取5 min作为衍生反应孵育时间。

#### 2.3.4 缓冲液pH

文献[19]报道2,2'-DTDP与游离巯基的衍生产物2-TP在pH 3.5~8.0可稳定存在,故本研究分别考察了pH为4、5、6、7、8的磷酸盐缓冲液对游离巯基衍生反应的影响。图3d显示pH值对衍生产物浓度影响不大。参考Riener等^[20]^关于pH对DTDP衍生反应影响的研究结果,最终选取pH 7的磷酸盐缓冲液。

#### 2.3.5 沉淀剂

充分衍生后的血清样本在进行HPLC分析前需要沉淀血清蛋白。本研究分别对比了甲醇、乙腈、异丙醇、高氯酸溶液、ZnSO_4_-甲醇混合溶液的沉淀效果。结果发现分别使用甲醇、乙腈和异丙醇沉淀蛋白后,样本体系均不能得到澄清透明的上清液,而使用高氯酸、ZnSO_4_-甲醇混合溶液作为沉淀剂,样本离心后可获得澄清透明上清液,色谱进样后峰形较好。考虑到高氯酸的强酸性和强氧化性,为避免引入未知副反应,本研究选择相对温和的ZnSO_4_-甲醇混合溶液作为沉淀剂。在此基础上,进一步比较了使用不同比例(4∶1、 3∶2、 1∶1、 2∶3、 1∶4, v/v)的1 mol/L ZnSO_4_-甲醇溶液沉淀体系的上清液时目标物的峰面积,结果显示ZnSO_4_-甲醇的体积比为1∶1时所得到的目标物色谱响应最佳、基线平稳、无明显杂质峰,故选用ZnSO_4_-甲醇(1∶1, v/v)混合溶液作为沉淀剂。

### 2.4 方法学验证

#### 2.4.1 特异性

血清样本加入2,2'-DTDP前后的色谱图如图2所示。在加入2,2'-DTDP前,血清样本未观察到明显的色谱峰(图2a)。当加入2,2'-DTDP后,除衍生剂自身色谱峰外,在2.3 min和2.8 min分别观测到两个明显的色谱峰(图2b),通过和游离巯基标准品衍生产物保留时间对比可以认定其分别为2-TP和LMM-S-S-Pyr。继续向血清样品中加入一定量的巯基标准品后,2-TP和LMM-S-S-Pyr色谱峰信号明显增强,同时没有新的色谱峰出现。以上结果表明只有血清巯基和衍生试剂同时存在才会产生目标产物色谱峰,证明本方法特异性好。

#### 2.4.2 线性关系、检出限、定量限

以半胱氨酸浓度为*X*、待测物色谱峰面积为*Y*进行线性回归分析。稀释标准溶液,分别以信噪比为3和10时的浓度作为检出限(LOD)和定量限(LOQ)。斜率、截距、相关系数(*R*)、线性范围、LOD、LOQ见表1。Total-SH和LMM-SH的5批次平均线性相关系数分别是0.9996和0.9994,线性良好。Total-SH和LMM-SH的检出限分别为2.61 和0.50 μmol/L,定量限分别为8.71 和1.67 μmol/L。

**表 1:** 待测物的斜率、截距、相关系数、线性范围、检出限、定量限(*n*=5)

Peak	Corresponding free thiol	Slope	Intercept	R	Linear range/ (μmol/L)	LOD/ (μmol/L)	LOQ/ (μmol/L)
2-TP	Total-SH	3058.66±40.29	-139.57±14.64	0.9996±0.0002	31.25-1000	2.61	8.71
LMM-S-S-Pyr	LMM-SH	4022.68±86.76	-138.12±39.01	0.9994±0.0002	31.25-1000	0.50	1.67

Total-SH: total free thiols; LMM-SH: low-molecular-mass free thiols.

#### 2.4.3 回收率和精密度

分别向两种血清样本中加入衍生剂和250、500 μmol/L的半胱氨酸标准溶液,每种加标样本平行制备3份,重复测定5个批次。测定加标前后的样本中游离巯基的含量,计算目标物的回收率及相对标准偏差(RSD),结果见表2。Total-SH的加标回收率为99.9%~105.4%,日内精密度和日间精密度RSD值为1.0%~7.6%, LMM-SH加标回收率为91.1%~106.0%,日内精密度和日间精密度RSD值为0.4%~9.1%。

**表 2:** 目标化合物在2个水平下的加标回收率和精密度

Peak	Corresponding free thiol	Sample	Background/ (μmol/L)	Added/ (μmol/L)	Detected/ (μmol/L)	Found^*^/ (μmol/L)	Recovery/ %(n=5)	Intra-day RSD/%(n=3)	Inter-day RSD/%(n=5)
2-TP	Total-SH	serum 1	459.52	250	712.24	252.72	101.1	1.3	4.5
				500	958.99	499.47	99.9	1.0	6.6
		serum 2	392.53	250	648.18	255.65	102.3	2.8	4.8
				500	919.60	527.07	105.4	2.9	7.6
LMM-S-S-Pyr	LMM-SH	serum 1	147.95	250	408.03	260.08	104.0	0.5	1.7
				500	603.38	455.43	91.1	0.5	9.1
		serum 2	110.94	250	375.86	264.92	106.0	1.0	2.3
				500	566.82	455.88	91.2	0.4	8.3

*Found=Detected-Background. Recovery=Found/Added ×100%.

### 2.5 方法的人群应用

为了进一步评价本研究方法的实用性,用所建立方法测定714位临床志愿者的血清Total-SH及LMM-SH含量,并计算P-SH含量。Total-SH、LMM-SH、P-SH的散点图如图4所示,可以看出志愿者人群的Total-SH浓度范围为376.60~781.12 μmol/L,平均浓度为555.62 μmol/L;LMM-SH浓度范围为36.37~231.65 μmol/L,平均浓度为82.34 μmol/L;P-SH含量为288.36~687.74 μmol/L,平均浓度为473.27 μmol/L。Total-SH与LMM-SH(*r*=0.200, *P*<0.001)、与P-SH(*r*=0.953, *P*<0.001)均呈显著的Spearman正相关趋势。

**图 4:**
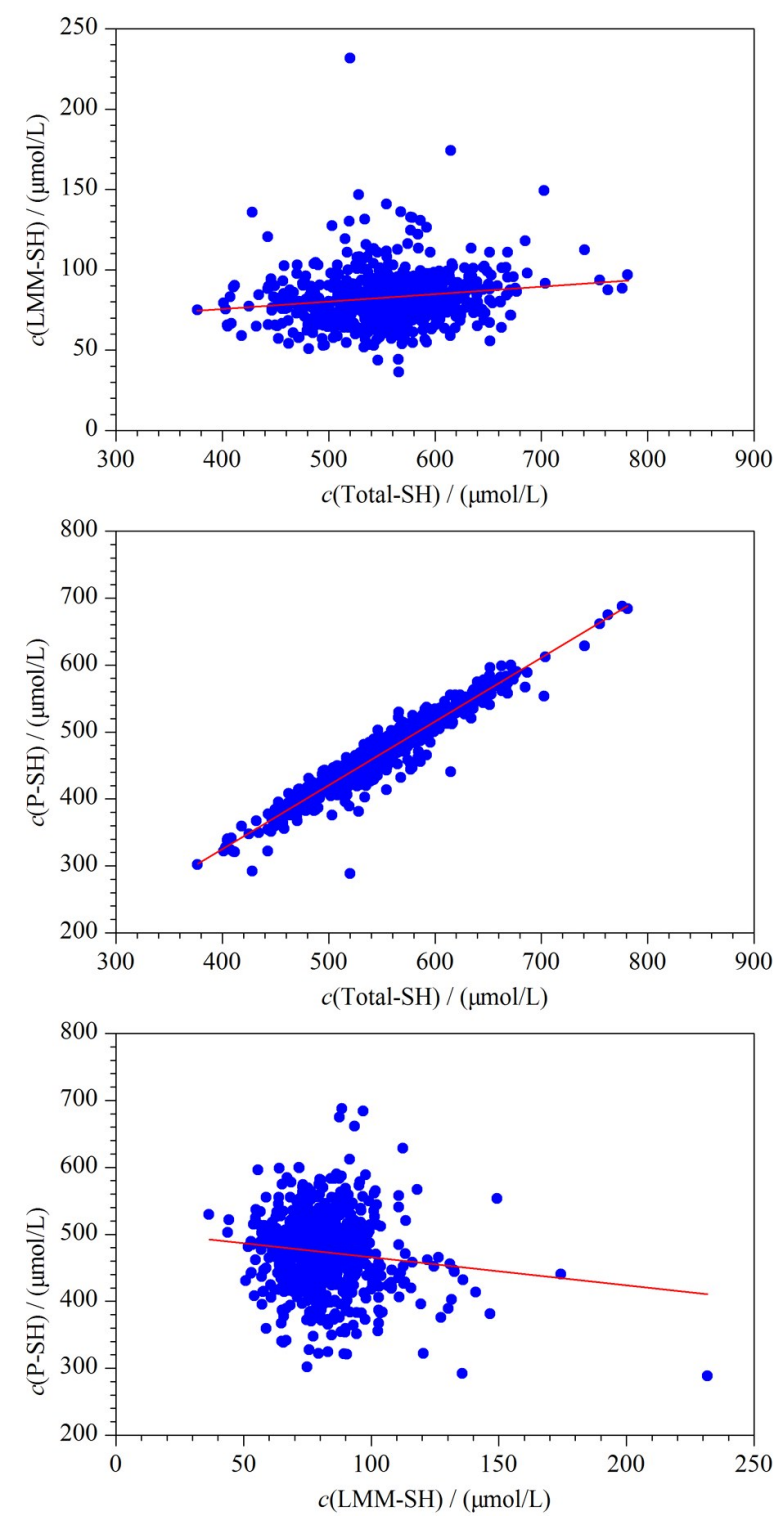
Total-SH、LMM-SH、P-SH的散点图

在此基础上使用非参数Spearman相关性分析,探索了Total-SH、LMM-SH、P-SH与冠心病相关临床指标之间的关系。结果如表3所示,Total-SH、LMM-SH、P-SH均与超氧化物歧化酶(SOD)呈统计学显著正相关且不受年龄和性别的影响,表明巯基与SOD一样具有抗氧化作用,可保护心血管系统免受氧化损伤。此外,Total-SH和P-SH与年龄、收缩压、超敏C反应蛋白(hsCRP)、氧化三甲胺(TMAO)、乳酸脱氢酶(LDH)、肌钙蛋白I(cTnI)、肌钙蛋白T(cTnT)、脑钠肽(BNP)、冠脉病变程度评分(Gensini评分)等临床指标均呈统计学负相关,特别是在校正了年龄、性别等混杂因素后,Total-SH和P-SH仍与炎症标志物(hsCRP)、动脉粥样硬化标志物(TMAO)、心肌损伤标志物(LDH、cTnI)、心衰标志物(BNP)以及Gensini评分等保持显著负相关。

**表 3:** 游离巯基与冠心病相关临床指标及严重程度分组的非参数Spearman相关系数

Variable	Total-SH	LMM-SH	P-SH
Gender	-0.014	0.079^*^	-0.034
Age	-0.481^***^	-0.026	-0.480^***^
SBP	-0.102^**^	-0.056	-0.095^*^
FBG	0.011	-0.033	0.023
TC	0.073	0.027	0.068
hsCRP	-0.117^*^	-0.044	-0.111^*^
SOD	0.856^***^	0.119^**^	0.831^***^
TMAO	-0.211^***^	-0.059	-0.200^***^
LDH	-0.239^***^	-0.001	-0.243^***^
BNP	-0.484^***^	-0.061	-0.473^***^
cTnI	-0.278^***^	-0.006	-0.275^***^
cTnT	-0.436^***^	-0.140	-0.447^***^
Gensini score	-0.185^***^	-0.016	-0.185^***^
CAD Classification	-0.096^*^	0.054	-0.117^**^

Gender (1: male; 2: female); SBP: systolic blood pressure; FBG: fasting blood glucose; TC: total cholesterol; hsCRP: high-sensitivity C-reactive protein; SOD: superoxide dismutase; TMAO: trimethylamine *N*-oxide; LDH: lactate dehydrogenase; BNP: brain natriuretic peptide; cTnI: cardiac troponin I; cTnT: cardiac troponin T; CAD Classification (1: Non-CAD; 2: SAP, stable angina pectoris; 3: ACS, acute coronary syndrome). * *P*<0.05; ** *P*<0.01; *** *P*<0.001.

心内科临床医师依据志愿者临床症状、体征、心电图、心肌酶学指标以及冠脉造影检查结果,综合评价,将其分别诊断为非冠心病(Non-CAD)、稳定型心绞痛(SAP)、不稳定型心绞痛、非ST段抬高型心肌梗死、ST段抬高型心肌梗死。其中后三者在临床合称为急性冠脉综合征(ACS)。本研究将志愿者人群中有明确临床诊断意见的703人,依据临床诊断结果,按照冠心病严重程度依次分为Non-CAD、SAP、ACS 3组。3组人群的巯基浓度分布及组间比较见图5,与Non-CAD组相比,SAP组和ACS组人群的Total-SH、P-SH含量均呈统计学显著性降低。这些结果都证明氧化应激状态与冠心病发生发展密切相关。同时也表明人血清游离巯基水平能够作为反映冠心病风险及疾病严重程度的客观指标,在冠心病临床预防和诊治过程中具有潜在的应用价值。

**图 5:**
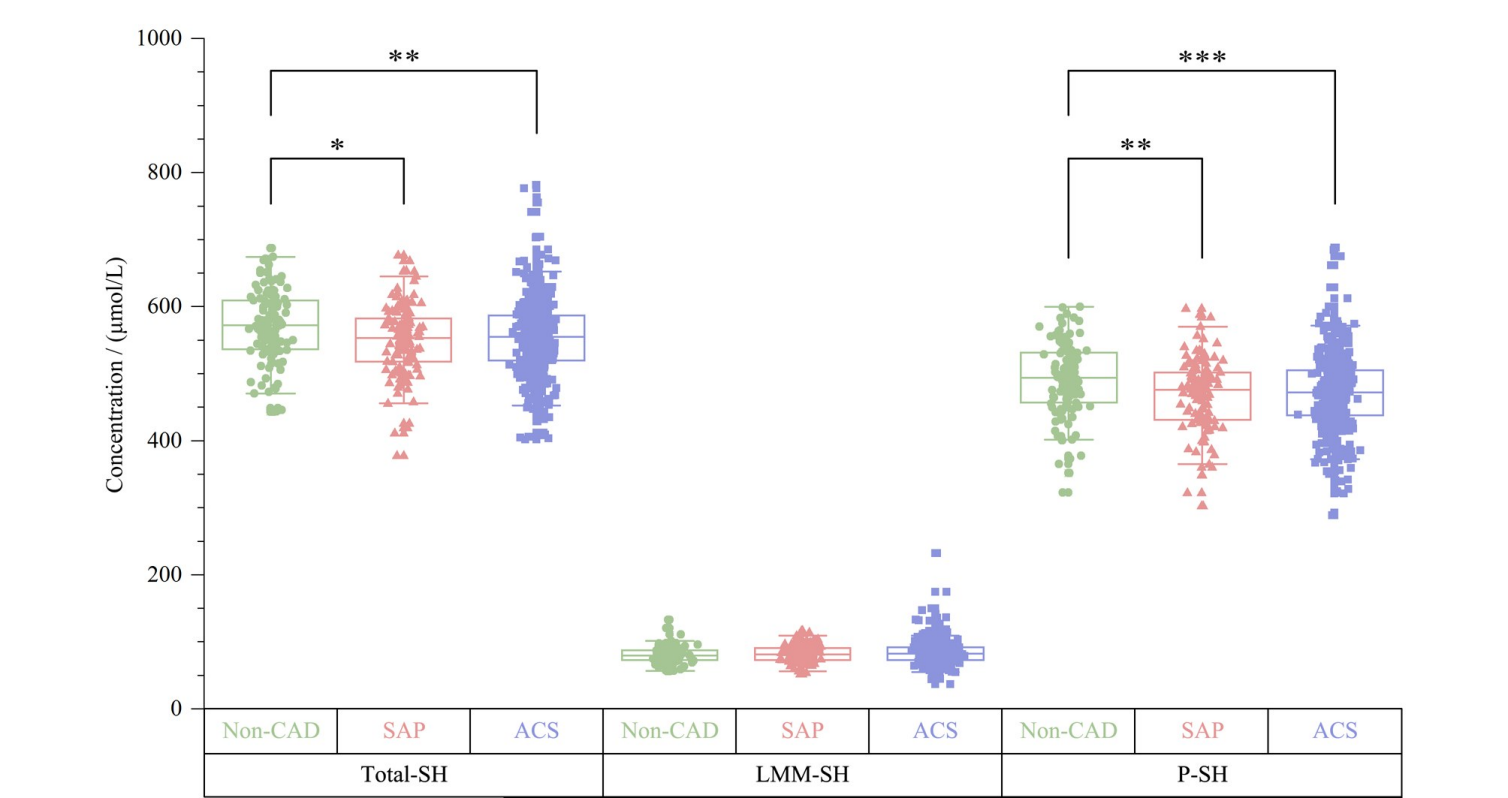
不同冠心病严重程度人群的Total-SH、LMM-SH、P-SH浓度比较

## 3 结论

本研究建立了一种高效液相色谱联合2,2'-DTDP衍生反应测定人血清游离巯基的分析方法。该方法快速、简便、精密,能够有效地分离和测定样本中的Total-SH和LMM-SH。一次进样即可同时获得血清中Total-SH、LMM-SH和P-SH的含量。该方法已证明可适用于大规模人群研究,为临床监测人群样本血清游离巯基水平提供了一个新方法,具有潜在的应用价值。本研究的人群分析结果为人血清游离巯基水平与冠心病之间的关系提供了新的参考依据,为血清游离巯基作为冠心病潜在生物学标志物的相关研究提供了新的数据支持。
